# Prevalence and Prescribers of Preoperative Opioid Prescriptions in the US, 2008-2019

**DOI:** 10.1001/jamanetworkopen.2021.47897

**Published:** 2022-02-10

**Authors:** Ryan Howard, Brooke Kenney, Chad Brummett, Jennifer Waljee, Michael Englesbe, Dana Telem

**Affiliations:** 1 Michigan Opioid Prescribing and Engagement Network, Institute for Healthcare Policy and Innovation, Ann Arbor; 2 Department of Surgery, University of Michigan, Ann Arbor; 3 Department of Anesthesiology, University of Michigan, Ann Arbor

## Abstract

This cross-sectional study examines the prevalence of preoperative opioid prescribing and the specialties of clinicians who prescribe these medications.

## Introduction

Patients may be prescribed opioids before surgical procedures to manage pain related to a surgical condition or to provide a postoperative prescription in advance. Filling an opioid prescription preoperatively is a risk factor for prolonged postoperative opioid use among patients who are opioid naive.^1,2,3^ However, efforts to reduce this risk have focused on decreasing opioid prescribing after surgical procedures.^4^ To better inform opioid stewardship, we examined preoperative opioid prescribing, which we hypothesized was common.

## Methods

This cross-sectional study was approved by the institutional review board at the University of Michigan. Informed consent was waived because data were deidentified. This study follows the Strengthening the Reporting of Observational Studies in Epidemiology (STROBE) reporting guideline.

We performed a retrospective review of Optum Clinformatics® Data Mart Database. Eligible patients were adults aged 18 years or older who were opioid naive and undergoing bariatric surgical procedures, cesarean delivery, carotid endarterectomy, colectomy, hemorrhoidectomy, hysterectomy, inguinal hernia repair, laparoscopic cholecystectomy, total hip arthroplasty, total knee arthroplasty, and ventral hernia repair from 2008 to 2019. Opioid naive status was established by excluding patients who filled an opioid prescription between 365 to 31 days before surgical procedures.^3^ The primary outcome was filling an opioid prescription before their surgical procedure (between preoperative days 30 to 1) among patients who filled a perioperative opioid prescription (between preoperative day 30 to postoperative day 3). Patients who filled a preoperative prescription were counted in the primary outcome even if they subsequently filled a postoperative opioid prescription.

The secondary outcome was the prescriber specialty for preoperative opioid prescriptions, identified using a taxonomy derived from National Provider Identifier.^4^ Specialties included surgical specialties; primary care physicians (PCPs); medical subspecialties; emergency medicine; physical medicine and rehabilitation (PM&R), pain, and addiction medicine specialists; dentists; other specialty types; and unknown if specialty was missing from the prescription.

Descriptive statistics were calculated for both outcomes. Data analysis was conducted from February 2021 to April 2021. The study flow diagram is in the eFigure in the Supplement.

## Results

This study included 1 342 402 patients who underwent surgical procedures during the study period. The mean (SD) age was 54.2 (18.0) years, 843 271 (62.8%) patients were women, and 499 131 (37.2%) were men. Among 863 460 patients who filled an opioid prescription, 118 330 patients (13.7%) filled a prescription in the 30 days before their surgical procedure, of whom the mean (SD) age was 53.7 (16.0) years and 76 667 (65.0%) were female. The frequency of filling a preoperative opioid prescription ranged from 28.6% for bariatric surgical procedures to 2.8% for cesarean delivery, and surgeons were the most frequent preoperative prescriber of opioids (45.9%) (Figure).

**Figure.**
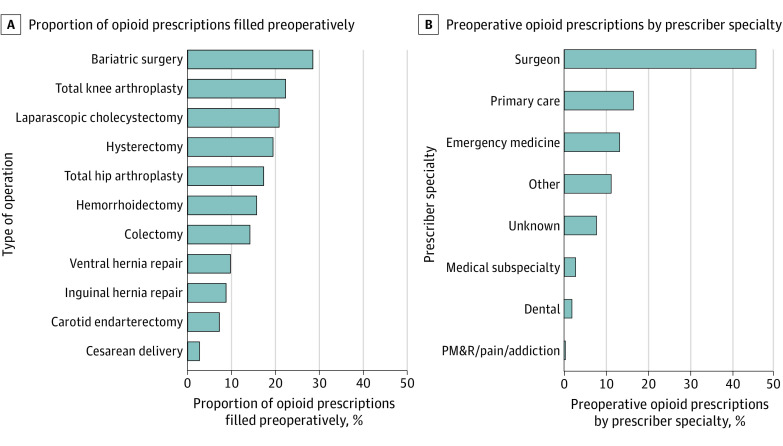
Proportion of Patients Who Filled a Preoperative Opioid Prescription by Procedure and Proportion of Preoperative Prescriptions Provided by Each Specialty Abbreviation: PM&R, physical medicine and rehabilitation.

The distribution of preoperative prescribers varied by procedure. Surgeons were the most frequent preoperative prescriber for 8 of the 11 procedures; however, PCPs were the most frequent prescriber before colectomy and carotid endarterectomy, and emergency medicine physicians were the most frequent prescriber before laparoscopic cholecystectomy (Table).

**Table.** Proportion of Preoperative Opioid Prescriptions Provided by Each Specialty for Each Procedure, in Order of Proportion Provided by Surgeons

Procedure	No. (%)							
	Surgeon	Primary Care	Emergency Medicine	Medical subspecialty	PM&R/pain/addiction	Dental	Other	Unknown
Hysterectomy	16591 (70.7)	1553 (6.6)	1012 (4.3)	304 (1.3)	50 (0.2)	310 (1.3)	2131 (9.1)	1517 (6.5)
Cesarian delivery	3789 (70.4)	232 (4.3)	127 (2.4)	74 (1.4)	3 (0.1)	120 (2.2)	500 (9.3)	538 (10.0)
Total knee arthroplasty	10290 (59.1)	2084 (12.0)	302 (1.7)	314 (1.8)	90 (0.5)	428 (2.5)	2891 (16.6)	1016 (5.8)
Bariatric surgery	3489 (59.0)	512 (8.7)	85 (1.4)	113 (1.9)	47 (0.8)	74 (1.3)	1089 (18.4)	507 (8.6)
Total hip arthroplasty	6848 (54.2)	2153 (17.0)	179 (1.4)	301 (2.4)	70 (0.6)	234 (1.9)	2064 (16.3)	791 (6.3)
Inguinal hernia repair	5675 (50.3)	1950 (17.3)	1121 (9.9)	255 (2.3)	41 (0.4)	310 (2.8)	1154 (10.2)	787 (7.0)
Ventral hernia repair	3836 (49.8)	1128 (14.6)	797 (10.3)	181 (2.4)	30 (0.4)	210 (2.7)	873 (11.3)	654 (8.5)
Hemorrhoidectomy	2608 (41.7)	1355 (21.7)	615 (9.8)	242 (3.9)	35 (0.6)	233 (3.7)	517 (8.3)	647 (10.4)
Colectomy	1747 (26.9)	1986 (30.6)	776 (12.0)	675 (10.4)	36 (0.6)	124 (1.9)	595 (9.2)	550 (8.5)
Carotid endarterectomy	182 (24.9)	233 (31.9)	68 (9.3)	58 (7.9)	12 (1.6)	47 (6.4)	80 (10.9)	51 (7.0)
Laparoscopic cholecystectomy	7316 (18.9)	9434 (24.4)	12996 (33.6)	1230 (3.2)	136 (0.4)	459 (1.2)	3471 (9.0)	3587 (9.3)

Abbreviation: PM&R, physical medicine and rehabilitation.

## Discussion

One in 7 patients who were opioid naive and filled a perioperative opioid prescription did so before their operation. Although the indication for prescriptions was not determined, the high prevalence of surgeon prescribers suggests prescriptions were related to the surgical procedures. A third of preoperative prescriptions were written by nonsurgical specialties and may also be intended to manage pain related to the surgical pathology or may be unrelated and simply coincidental to the timing of surgical procedures. Either way, previous studies^1,2^ show that preoperative prescriptions place opioid-naive patients at up to 5-fold greater risk of developing prolonged opioid use after surgical procedures. These results suggest that curbing excessive perioperative opioid prescribing will require multidisciplinary coordination.^4^

This study is limited by its retrospective nature, which introduces selection bias, and its reliance on administrative prescription claims, which are distinct from, although correlated with, actual opioid use.^5^ Moreover, patients who fill prescriptions preemptively may not use them. This study cannot ascertain the indication for each prescription, although excluding patients with prior opioid fills likely precludes many chronic pain conditions. Overall, these results suggest that preoperative opioid prescribing may be prevalent and that opportunities for practice improvement exist across specialties. Future work should explore the association between preoperative opioid exposure and postoperative outcomes.

## Notes

Supplement. **eFigure.** CONSORT Diagram
