# Supplementary material for: Prevalence and Prescribers of Preoperative Opioid Prescriptions in the US, 2008-2019
Source: JAMA Netw Open. 2022 Feb 10;5(2):e2147897. doi: 10.1001/jamanetworkopen.2021.47897 (PMC8832172; doi:10.1001/jamanetworkopen.2021.47897)
Supplement: Supplement. — eFigure. CONSORT Diagram [file jamanetwopen-e2147897-s001.pdf]

Supplemental Online Content

Howard R, Kenney B, Brummett C, Waljee J, Englesbe M, Telem D. Prevalence and prescribers of preoperative opioid prescriptions in the US, 2008-2019. *JAMA Netw Open*. 2022;5(2):e2147897. doi:10.1001/jamanetworkopen.2021.47897

eFigure. CONSORT Diagram

This supplemental material has been provided by the authors to give readers additional information about their work.

© 2022 Howard R et al. *JAMA Network Open.*

eFigure. CONSORT Diagram

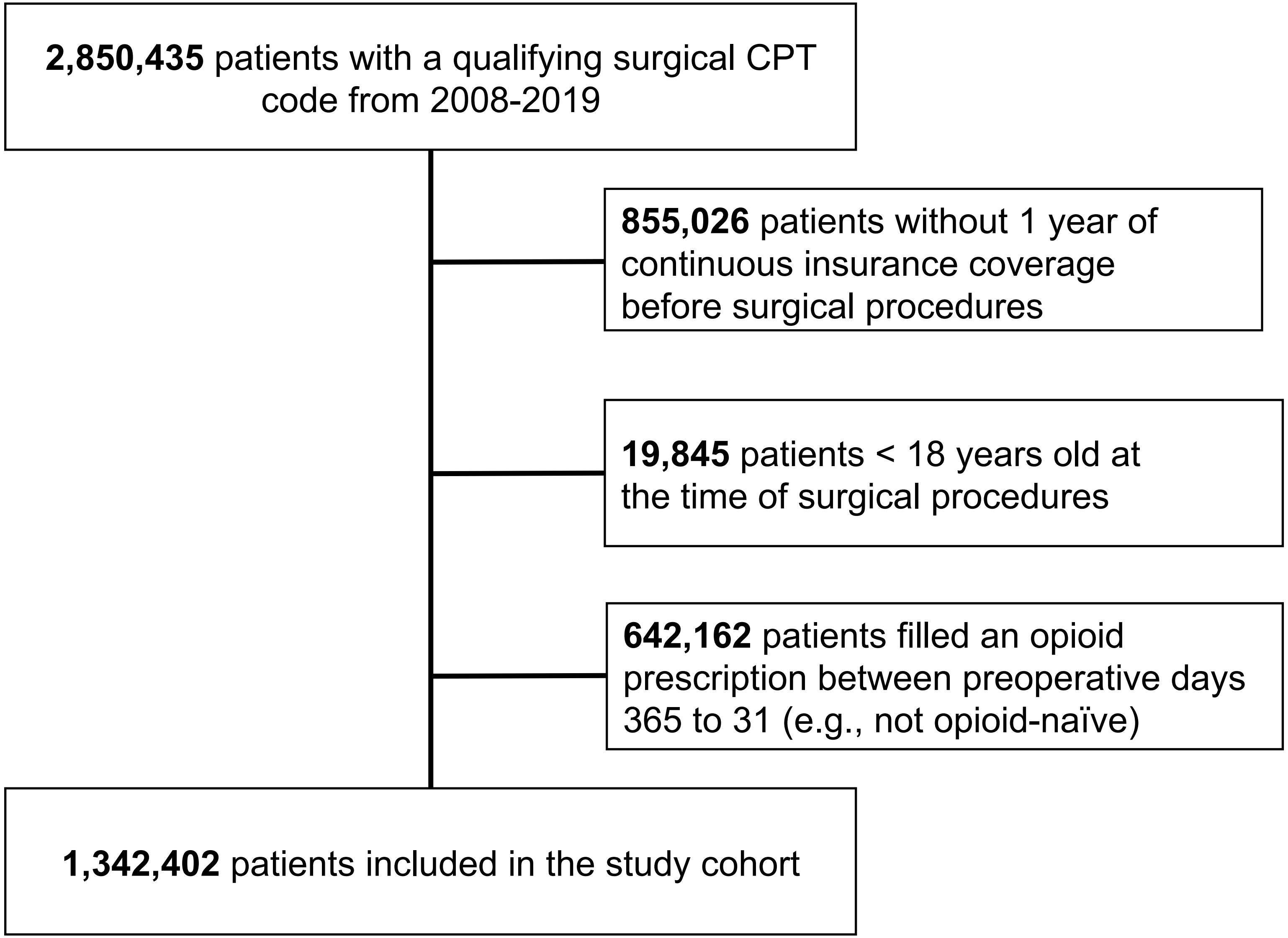

© 2022 Howard R et al. *JAMA Network Open*.
